# Evidence-Based Behavioral Strategies in Smartphone Apps for Children’s Sleep: Content Analysis

**DOI:** 10.2196/32129

**Published:** 2022-03-03

**Authors:** Stacey L Simon, Jill L Kaar, Ishaah Talker, Jennifer Reich

**Affiliations:** 1 Department of Pediatrics University of Colorado Anschutz Medical Campus Aurora, CO United States; 2 Department of Sociology University of Colorado Denver Denver, CO United States

**Keywords:** pediatrics, technology, smartphones, health behavior, sleep applications, children, mobile health, mHealth, smartphone applications, health applications, sleep disorders, sleep problems, developer descriptions, apps

## Abstract

**Background:**

Empirically supported treatments for pediatric sleep problems exist, but many families turn to other sources for help with their children’s sleep, such as smartphone apps. Sleep apps are easy for families to access, but little evidence exists regarding the validity of the services and information provided in the developer descriptions of the apps.

**Objective:**

The goal of this study was to examine the features and claims of developer descriptions of sleep apps for children.

**Methods:**

A search of the Apple iTunes store and Google Play was conducted using the terms “kids sleep,” “child sleep,” and “baby sleep.” Data on the type of app, price, user rating, and number of users were collected. Apps were analyzed in comparison with evidence-based behavioral strategies and were thematically coded on the basis of claims provided in developer descriptions.

**Results:**

A total of 83 app descriptions were examined, of which only 2 (2.4%) offered sleep improvement strategies. The majority were sound and light apps (78%) and 19% were bedtime games or stories. Only 18 of 83 (21.6%) apps were identified as containing empirically supported behavioral sleep strategies. Despite this, many apps asserted claims that they will help children “fall asleep instantly,” “cry less and sleep better,” or improve child development.

**Conclusions:**

A large variety of sleep apps exist for use among children, but few include evidence-based behavioral strategies according to the developer descriptions of the apps. Addressing sleep difficulties in children is important to promote physical, cognitive, and emotional development. Collaboration between sleep researchers and technology developers may be beneficial for creating evidence-supported apps to help with children’s sleep in the future.

## Introduction

Sleep problems in young children are common and associated with significant negative behavioral and physical consequences for children as well as increased sleep disruption and stress for their parents [1]. Approximately 20%-30% of infants, toddlers, and children have significant difficulties with falling asleep and night wakings, and pediatric sleep difficulties are among the most common complaints reported by parents to pediatricians [2,3]. Empirically supported treatments for pediatric sleep problems exist, but many families face barriers in seeking appropriate care, such as difficulty accessing a provider with specialized sleep training [4,5]. In particular, pediatricians may lack knowledge about appropriate sleep interventions for children [5]. Hence, many parents may turn to other sources for help with their children’s sleep, including technological strategies such as smartphone apps.

While no studies have previously examined sleep apps for children, 2 studies have examined sleep apps for adults. One study examined behavioral constructs contained within the apps to evaluate how well these apps are grounded in behavioral theory, which has a strong evidence base for sleep interventions [6]. Grigsby-Toussaint et al [6] evaluated 35 sleep apps for adults and found that only 34% incorporated evidence-based behavioral constructs. The most common behavioral constructs were realistic goal setting, time management, and self-monitoring. Authors also found a positive but nonsignificant association between higher user rating of the app and number of behavioral constructs. Another study examined empirical evidence contained within the developer descriptions of sleep apps targeted to adults from Google Play and found that only 33% of sleep apps contained empirical evidence to support claims made in the app descriptions [7]. The most common empirical evidence provided was information on how sleep is affected by drugs and alcohol (24%), food (13%), daily activities (13%), and stress (13%). User ratings were higher for the apps containing at least one source of empirical information compared to those without empirical information. However, user ratings were also higher for apps that contained a “sleep tip” function, regardless of whether these tips were based on empirical evidence. Thus, sleep apps available on the market may not be grounded in behavioral constructs or contain evidence-based information, but this has not yet been examined for apps aimed at children.

Sleep apps are easy for families to access given today’s high rates of smart phone usage and mobile internet availability [8], but little evidence exists about the sleep apps available for children, or the validity of the services and information provided in the developer description of the apps. Because families may search for these apps independently (eg, without support of a health care professional), it is essential that the app descriptions contain accurate information. Thus, the goal of this study was to (1) examine the number and characteristics of sleep apps for children and (2) analyze the purported features and claims in the developer description of these apps. We hypothesized that a large number of sleep apps for children would exist, but that few would describe evidence-based behavioral strategies.

## Methods

**Figure 1:**
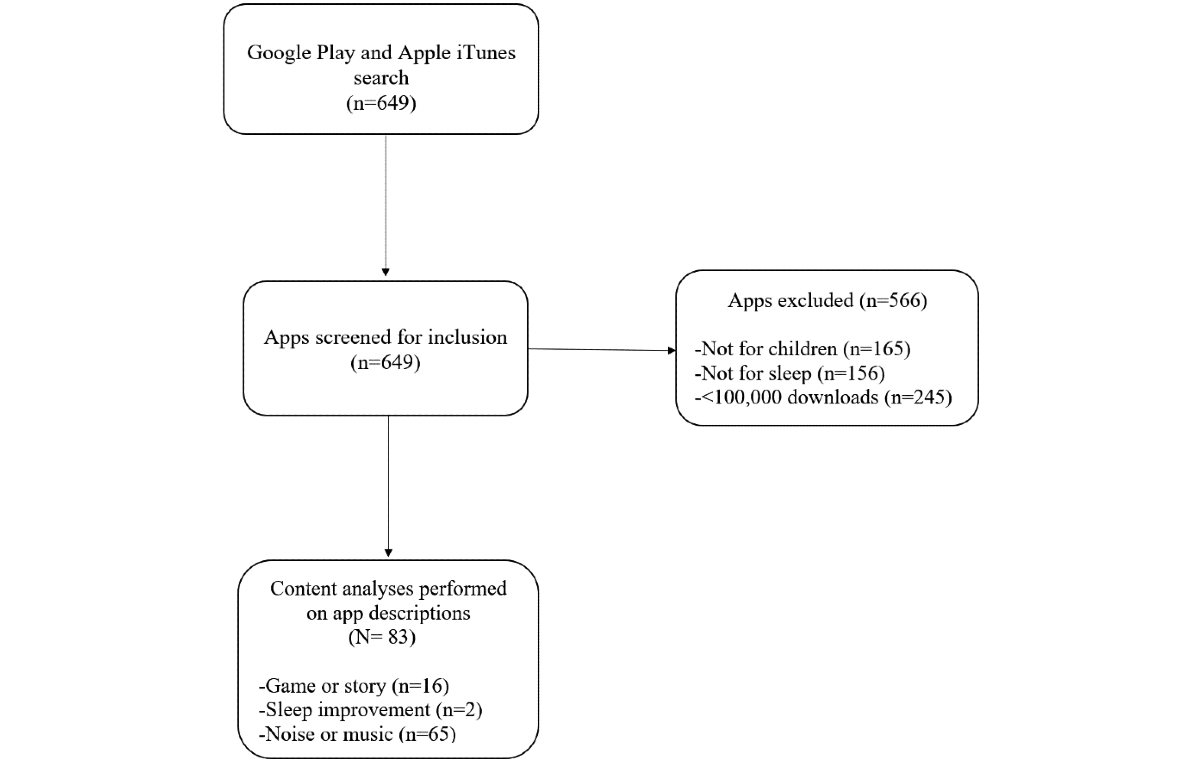
Flow diagram for the app search in this study.

An English language search of the Apple iTunes store and Google Play was conducted in December 2019, using the terms “kids sleep,” “child sleep,” and “baby sleep.” A total of 649 apps were initially identified. Apps were excluded if they were not specifically for children (n=165) or not for sleep (n=156). To focus analysis on apps that are actually used by parents, apps that had <100,000 downloads (n=245) were also excluded from the analysis. Figure 1 shows a flow chart of the app search.

Data on the type of app, price, user rating, and number of users were collected. The developer descriptions of the apps were analyzed in terms of comparison to evidence-based behavioral strategies, using a checklist of evidence-based behavioral strategies derived from the ABC’s of SLEEPING pediatric sleep recommendations [9]. Recommendations that received a rating of Strong or Moderate support, defined as support for the recommendation from at least 3 studies from well-designed studies without clearly contradicting findings, were included [9]. Inclusion of each strategy within the app description was coded as 0 (not present) or 1 (present). The specific strategies included are outlined in Table 1. Additionally, descriptions were thematically coded to identify patterns in these claims [10]. Codes were generated by reading the descriptions and generating a list of claims made by app developers. Data were coded by author IT and cross-checked by first author SLS. If there was a discrepancy in the coded data, all authors discussed and agreed upon the final data.

**Table 1:** Evidence-based behavioral strategies described in pediatric sleep apps^a^.

Behavioral strategies	Apps, n (%)	App name	Example description
Sufficient sleep opportunity for age	1 (1)	Huckleberry: Baby & Child Tracker^b^	“Tailored sleep schedules taking into account your child’s sleep needs”
Bedtime no later than 9 PM	0 (0)	N/A^c^	N/A
Consistent sleep schedule	2 (2)	Huckleberry: Baby & Child Tracker^b^ JOHNSON’S BEDTIME Baby Sleep	“Tracks your child’s day-to-day schedule to assist with setting bed and wake times”
Bedtime routines	4 (5)	Goodnight My Baby Huckleberry: Baby & Child Tracker JOHNSON’S BEDTIME Baby Sleep^b^ Moshi Twilight Sleep Stories: Kids Bedtime App	“Promotes a 3-step nighttime routine to help baby fall asleep and sleep through night”
Limited access to electronics during and after bedtime	0 (0)	N/A	N/A
Positivity and relaxation to help transition to sleep	16 (19)	Baby Lullabies Baby Mozart Effect Baby Sleep Baby Sleep Lullabies Baby Sleep Lullaby Music Box Baby Sleep Music 2019 Baby Sleep Sounds White Noise Baby Sleep: White Noise Baby Sleeping Music Baby Songs (Bipfun) JOHNSON’S BEDTIME Lullaby for Babies Lullaby for babies (desenvdroid) Moshi Twilight Sleep Stories: Kids Bedtime App^b^ Music Box to sleep Sleeptot: Baby White noise	“Audio plays to calm children prior to bedtime to prepare them to fall asleep”
Independent sleep skill development	0 (0)	N/A	N/A
Emotional needs met during the day	0 (0)	N/A	N/A

^a^A total of 18 out of 83 (21%) sleep apps had at least one strategy.

^b^App from which the example description was taken.

^c^N/A: not applicable.

## Results

### Results Overview

A total of 83 app descriptions were examined. Only 2% (2/83) specifically claimed to offer sleep improvement strategies, while the majority (78%, 65/83) were white noise or music apps, and 19% (16/83) were bedtime games or stories. The apps were highly rated (average 4.4; range 1-5, with 5 being the most favorable rating) and most were free (65%, 54/83); the price of paid apps ranged from US $0.99-$239.99 (annual subscription). Table 2 contains a complete list of the characteristics of all of the apps examined*.*

**Table 2:** Characteristics of sleep apps for children.

App name			Price (US $)		Rating		Users, n		Themes		Behavioral strategies
**Apps with sleep improvement strategies**											
	Huckleberry: Baby & Child Tracker, Sleep Experts	119.99		4.80		>100,000		Good sleep habits		Sleep opportunity, Sleep Schedule, and routines	
	JOHNSON’S BEDTIME Baby Sleep	0.00		3.70		>100,000		Help fall asleep and good sleep habits		Sleep schedule, routines, and relaxation	
**White noise or music apps**											
	Baby Lullabies	0.00		4.50		>100,000		Stop crying		Relaxation	
	Baby Lullaby Songs to Sleep^a^	0.00		4.00		>500,000		Help falling asleep and good sleep habits		None	
	Baby Lyrics & Songs	2.99		4.50		>500,000		Help falling asleep		None	
	Baby Mozart Effect^b^	0.00		4.40		>100,000		Well-being and help falling asleep		Relaxation	
	Baby Night Light: Instant Sleep Aids & White Noises^b^	3.49		4.40		>1,000,000		None		None	
	Baby Sleep^b^	0.00		4.70		>500,000		Well-being and help falling asleep		Relaxation	
	Baby Sleep Instant^b^	1.00		3.80		>100,000		Stop crying		None	
	Baby Sleep Lullabies^a^	0.00		4.10		>1,000,000		Help falling asleep		Relaxation	
	Baby Sleep Lullaby Music Box	5.99		4.70		>500,000		Well-being		Relaxation	
	Baby Sleep music (leopfinpamcev)^b^	0.00		4.00		>100,000		Help falling asleep		None	
	Baby Sleep Music 2019^b^	0.00		4.40		>500,000		Stop crying		Relaxation	
	Baby Sleep Sounds	0.00		4.80		>500,000		Help falling asleep		None	
	Baby Sleep Sounds- Sleep Sounds for Baby^b^	0.00		4.30		>100,000		None		None	
	Baby Sleep Sounds White Noise	0.99		4.10		>500,000		Help falling asleep		Relaxation	
	Baby Sleep: White Noise	3.99		4.80		>1,000,000		Stop crying		Relaxation	
	Baby Sleep: White Noise Lullabies for Newborns^b^	0.00		4.70		>1,000,000		Help falling asleep and trusted by parents		None	
	Baby Sleeping Music^b^	0.00		4.70		>100,000		Well-being and help falling asleep		Relaxation	
	Baby Sleeping Music (Free)^b^	0.00		4.10		>500,000		Help falling asleep		None	
	Baby Songs^b^	0.00		4.50		>1,000,000		Help falling asleep and good sleep habits		None	
	Baby Songs & Lullaby: Sounds for Bedtime & Naptime^b^	0.99-3.59		3.80		>100,000		None		None	
	Baby Songs (Bipfun)	3.59		3.80		>100,000		Well-being and help falling asleep		Relaxation	
	Baby Stop Crying and Sleep^b^	0.00		3.90		>500,000		None		None	
	Bedtime Music Lullaby Songs^b^	5.99		4.70		>100,000		None		None	
	Brahm's Lullaby for Babies^b^	0.00		4.70		>100,000		None		None	
	Calming music for kids to go to sleep^b^	0.00		4.20		>1,000,000		Well-being and help falling		None	
	Calms Baby with Womb Sound^b^	0.00		4.20		>100,000		None		None	
	Children Sleep Songs^b^	0.00		4.10		>1,000,000		None		None	
	Classical Music for Baby^b^	9.99		4.80		>100,000		None		None	
	Colic Baby-Baby Sleeping Sound^b^	0.00		4.60		>100,000		Stop crying		None	
	Don’t Cry My Baby (Lullaby)	0.00		4.60		>1,000,000		Stop crying, well-being, help falling asleep		None	
	Hair Dryer Sounds^b^	0.00		4.50		>100,000		Help falling asleep		None	
	Hatch Baby Rest	0.00^c^		4.60		>100,000		None		None	
	iWhite Noise Baby Bedtime Sound^b^	0.00		4.40		>100,000		None		None	
	Kids Sleep Songs Free^b^	0.00		4.20		>1,000,000		None		None	
	Lullabies Relax & Sleep Baby^b^	3.99		4.70		>1,000,000		Help falling asleep		None	
	Lullabo: Lullaby for Babies^b^	3.99		4.80		>100,000		Help falling asleep		None	
	Lullaby for Babies (dream_studio)^b^	0.00		4.80		>1,000,000		Well-being and help falling asleep		Relaxation	
	Lullaby for babies (desenvdroid)^b^	0.00		4.80		>5,000,000		Well-being and help falling asleep		Relaxation	
	Lullaby for Babies (desenvemax)^b^	0.00		4.60		>100,000		Help falling asleep		None	
	Lullaby for Babies 2^b^	0.00		4.80		>1,000,000		Help falling asleep		None	
	Lullaby for babies offline^b^	0.00		4.70		>100,000		Help falling asleep		None	
	Lullaby for Baby	7.99		4.70		>500,000		None		None	
	Lullaby Sleep Music for Babies^b^	0.00		4.60		>100,000		None		None	
	Lullaby Songs for Baby	9.99		4.60		>100,000		Help falling asleep		None	
	Lullaby Songs- Relax Music for Baby Sleep Light^b^	1.99		4.80		>100,000		None		None	
	Mozart Baby Sleep^b^	1.99		4.40		>100,000		Well-being		None	
	Music Box to sleep^b^	0.00		4.80		>1,000,000		Stop crying		Relaxation	
	Night Light	0.00		4.40		>500,000		Help falling asleep		None	
	Pinkfong Bedtime	9.99		4.40		>500,000		Help falling asleep		None	
	Sleep Baby Sleep	0.00		4.50		>100,000		Help falling asleep		None	
	Sleep Baby Sweet Dreams^a^	0.99		4.50		>100,000		Help falling asleep		None	
	Sleep Cute Baby Lullaby	0.99		4.50		>100,000		None		None	
	Sleeptot: Baby White Noise	28.99		4.50		>1,000,000		Well-being, help falling asleep, and trusted by parents		Relaxation	
	Sleepy Sounds	0.00		4.30		>1,000,000		Help falling asleep		None	
	Sound Sleeper	15.99		4.90		>100,000		Good sleep habits		None	
	Sound to Children Sleep	0.00		4.70		>1,000,000		Well-being, help falling asleep, and trusted by parents		None	
	Sounds for Baby Sleep Music^b^	0.00		4.70		>1,000,000		Help falling asleep		None	
	Sweet Dreams- Baby Songs^b^	0.00		3.90		>100,000		Help falling asleep		None	
	Sweet Lullabies ~Voice & Piano^b^	0.00		4.30		>100,000		None		None	
	White Noise & Deep Sleep Sounds- Fan & Baby Sleep^b^	19.99		4.50		>100,000		None		None	
	White Noise Baby	0.99		4.80		>1,000,000		Stop crying and well-being		None	
	White Noise: Baby Sleep Sounds	0.00		4.60		>100,000		Well-being		None	
	White Noise Baby Sleep Sounds^b^	0.00		4.80		>1,000,000		Help falling asleep and trusted by parents		None	
	White Noise for Baby^b^	0.00		4.80		>100,000		Well-being		None	
	White Noise: Baby Sleep & Lullaby Songs Calm & Nap^b^	1.98		4.90		>100,000		None		None	
**Bedtime games and story apps**											
	Bedtime Stories for Children- Story Books to read^b^	1.99-19.99		4.40		>100,000		None		None	
	Bedtime Stories for Kids	2.49		4.30		>1,000,000		Well-being and help falling asleep		None	
	Bedtime Stories Goodnight: short stories	0.00		3.90		>100,000		Good sleep habits		None	
	Best Kids Stories: bedtime^b^	38.99		4.40		>1,000,000		None		None	
	Children's Songs Lullabies^b^	5.99		4.80		>100,000		None		None	
	Good Night Hippo	1.99		4.40		>1,000,000		None		None	
	Goodnight Caillou	6.99		4.10		>5,000,000		None		None	
	Goodnight, My Baby^b^	0.00		4.10		>1,000,000		Well-being and good sleep habits		Routines	
	Kids Bedtime Stories- Fairy Tales^b^	3.49		4.10		>100,000		None		None	
	Little Stories: Read Bedtime Story Books For Kids	13.99		4.30		>100,000		Well-being		None	
	Lullabies and Bedtime Stories^b^	0.00		3.80		>100,000		None		None	
	Masha and the bear: good night	5.49		3.70		>5,000,000		None		None	
	Moshi Twilight Sleep Stories: Kids Bedtime App	239.99		4.00		>500,000		Help falling asleep and trusted by parents		Routines and relaxation	
	Nighty Night- Bedtime Story	1.99		4.60		>500,000		None		None	
	Storybook- Bedtime Stories & Baby Sleep Massage	5.99-47.99		2.70		>100,000		None		None	
	Teddy Bears Bedtime Stories	3.49		4.50		>500,000		Help falling asleep		None	

^a^Only available on the Apple App store.

^b^Only available on the Google Play store.

^c^Requires purchase of device.

### Types of Apps

#### Sleep Improvement Strategy Apps

Sleep improvement strategies apps (n=2) are both designed for parents of young children and contain parenting advice alongside sleep logs that allow users to track their children’s sleep patterns. Both of the sleep improvement apps have recommendations from sleep experts and guided steps for how to help children fall asleep. The *Huckleberry: Baby & Child Tracker, Sleep Experts* app is described as offering “an all-star team of sleep experts, personalized analysis and personalized step-by-step guidance of a traditional sleep consultant with the convenience of an app.” The sleep experts reportedly include nurse practitioners, certified sleep consultants, and board-certified behavioral therapists. For a fee, users can log their children’s sleep schedule and receive an analysis and recommendations. The *JOHNSON’S BEDTIME Baby Sleep* app states that it “answers your sleep related questions, gives advice and helps track and learn your baby’s sleep habits.” It recommends a 3-step bedtime routine consisting of bath, massage, and quiet time, which states has been tested in infants 7 months of age and older for at least 1 week of use. While not cited in the description of the app, the *JOHNSON’S BEDTIME* app indeed has published data supporting these claims: a trial of over 400 infants (mean age 8.3 months) found that parents reported increased sleep duration and improved sleep quality after use of the app [11].

#### White Noise or Music Apps

White noise or music apps (n=65) feature music or various sounds that are intended to be played during the night to help children sleep better. Most of the apps appear targeted for use with infants, with 45 of 83 (69%) containing the word “baby” in the app name. Two of the apps (3%) specify that they have timers to shut off the sounds after a predetermined time, while 5 apps (8%) have the ability to play sounds continuously; the remainder did not specify the duration or timing features of the sounds. In addition to playing music and sounds, the *Baby Night Light – Sleep Aid* app features sound detection such that if the app hears noises in the room, it will automatically turn on a nightlight to “soothe and put your child to sleep again when a baby wakes up.” In contrast, the *White Noise Baby* app features “looped ambient sounds and music,” allowing it to be played and maintained the entire night. These apps do not offer evidence of efficacy.

#### Bedtime Games or Story Apps

The apps featuring games and stories (n=16) feature animals or other creatures going through a bedtime routine, becoming sleepy, and falling asleep, accompanied by music. All apps encourage parents to use the app with their toddler, preschool, or school-aged child as part of a nightly routine. For example, the *Nighty Night!* app is described as a “daily go-to-sleep ritual with cute animals, sweet lullaby music, and great narration.” Some apps indicate they should be used simultaneously while children attempt to fall asleep, such as the *Sweet Dreams: Good Night Books* app which states, “[the animals] all fall asleep and so will do [*sic*] your little one at the end of the app.” Only one app, *Moshi Twilight Sleep Stories: Kids Bedtime App*, utilized audio-only stories, meditations, music and sounds to help “settle and soothe kids into peaceful and restful sleep.”

### Themes From Content Analysis

Several themes emerged from the descriptions of the apps, including the common claim that the app has the ability to help children fall asleep quickly, improve child well-being and development, stop children from crying, help children develop good sleeping habits, and are trusted by parents.

#### Helps Children Fall Asleep

Many of the apps purported to be able to help children fall asleep quickly and easily (38/83, 46%). The *Sleep Baby Sweet Dreams* app stated, “the app will help you put your infant children to sleep quickly and calmly,” while the *Lullaby for Baby* app stated “children fall asleep immediately” with its use. None of the app descriptions explained the mechanisms by which the app will accomplish this nor cited evidence for this statement.

#### Improve Well-being and Development

Another theme was that use of the app would improve the well-being or development of children (18/83, 22%). *Mozart Baby Sleep* stated it will help babies “brain development, memory stimulation, and positive emotions.” The *Little Stories: Read Bedtime Story Books for Kids* app stated, “these stories have a positive impact on the development of your child.” Similarly, the *Baby Mozart Effect* app claimed that it “quickly helps calm your baby, reduces the stress of new life, enhances auditory and emotional awareness, induces relaxation and sleep.” However, none of these claims of supporting well-being and development were backed with evidence.

#### Stop Crying

One common theme was a claim that the app can stop children from crying at bedtime or at night (8/83, 9%). The *White Noise Baby* app stated that it will “help your baby relax, stop crying, and sleep better.” Similarly, *Baby Lullabies* stated that its “natural white noise and soothing sounds helps babies cry less and sleep better.” However, none of the apps making this statement addressed evidence-based behavioral management strategies to help children learn self-soothing strategies to fall asleep independently.

#### Develop Good Sleeping Habits

One theme of the apps was that they could help children develop positive sleeping habits and routines, often through use of games, stories, or songs (7/83, 8%). The *Goodnight, My Baby* app “let[s] your children develop a good sleeping habit when they encourage their friends to do the same.” The *Baby Songs* music app states, “with these wonderful tunes, your baby will establish a healthy bedtime routine!” Most of the apps did not provide support or describe how the app would accomplish sleep routine development. However, both the *Huckleberry: Baby & Child Tracker, Sleep Experts* app and the *JOHNSON’S BEDTIME Baby Sleep* app reported empirical support and use of behavioral strategies to improve child sleep habits. The *Huckleberry* app stated users can “access guidance from pediatric sleep experts, and daily personalized sleep plans for your child.” The *JOHNSON’S* app includes a “3-step nighttime routine, the only one that has been clinically proven to help baby fall asleep faster and sleep through the night better.”

#### Trusted by Parents

Several apps implied that they should be used because they are endorsed by parents (5/83, 6%). The *White Noise Baby Sleep Sounds* app stated it has been “proven to be effective by generations of parents.” The *Moshi Twilight Sleep Stories: Kids Bedtime App* claims that “97% of parents surveyed agree it helps get their kids to sleep quicker, 95% say makes bedtime less stressful.” No information on survey methodology or citations were provided for these claims.

### Behavioral Strategies

In total, 18 (21.6%) apps were found to contain at least one evidence-based behavioral sleep strategy, most commonly relaxation (16/83, 19.3%). Table 1 includes the behavioral strategies described in the apps. None of the descriptions of the apps explicitly included strategies such as bedtime no later than 9 PM, limiting access to electronics during and after bedtime, independent sleep skill development, or meeting emotional needs during the day. Three of the apps included more than one behavioral strategy: the *JOHNSON’S BEDTIME Baby Sleep* app included both bedtime routines and a consistent sleep scheduling, the *Moshi Twilight Sleep Stories: Kids Bedtime App* included relaxation and bedtime routines, while the *Huckleberry: Baby & Child Tracker, Sleep Experts* app included four strategies (relaxation, bedtime routines, consistent sleep scheduling, and sufficient sleep opportunity for age). The *JOHNSON’S BEDTIME Baby Sleep* app is the only app found to have supportive evidence from a nonrandomized real-world effectiveness trial [11].

## Discussion

### Principal Findings

Over 80 sleep apps were analyzed, which were created for the purpose of improving a child’s sleep, each downloaded more than 100,000 times. Most of these are apps that purport to offer white noise or soothing music but do not actually address sleep habits specifically. Several themes emerged from the developer descriptions of the apps, including the ability to help children fall asleep quickly, improve well-being and development, stop children from crying, help develop good sleeping habits, and that the apps are trusted by parents. The majority of apps did not include evidence-based behavioral strategies for sleep in their description or claims. The apps that did include behavioral strategies mentioned the use of relaxation, consistent sleep scheduling, bedtime routines, and allowing sufficient sleep opportunity for age.

Overall, our findings show that apps targeting sleep in pediatric populations were less likely to incorporate evidence-based behavioral strategies than sleep apps targeted to adult populations (only 21.6% vs 33%-34%) [6,7]. Our findings are consistent with a lack of evidence-based support in apps for other childhood difficulties, such as apps for infant feeding [12,13]. Of note, an app may have promoted one evidence-based strategy while simultaneously being in contradiction of another; for example, many of the white noise or music apps and bedtime games or stories apps stated they could be used for relaxation at bedtime, and they appeared to be intended for use visually during the bedtime routine (in opposition to the recommendation to limit electronics during or after bedtime) [9,14]. Using an app while children are falling asleep could create a sleep onset association such that children may then not be able to fall asleep independently without utilizing electronic devices [2].

However, 3 apps contained more than one evidence-based behavioral sleep strategy. As our analysis was based solely on the app description, it is possible that the content within the app may have indicated even more of these strategies. These findings suggest that apps can be developed, which are in line with the evidence base for pediatric sleep. Unfortunately, the majority of currently available sleep apps may not be a good source of evidence-based behavioral strategies for pediatric sleep problems. Moreover, the *JOHNSON’S BEDTIME Baby Sleep* app was the only app with support from a real-world effectiveness trial [11]. Future research examining the efficacy and effectiveness of sleep apps for pediatric sleep problems is recommended.

### Strengths and Limitations

Sleep apps targeted at improving children’s sleep have room for improvement regarding input from the scientific and clinical community. To our knowledge, this is the first review of such apps, and although a systematic approach was followed to assess each apps content, this analysis does have limitations. The current examination was for apps found with the search terms “kids sleep,” “child sleep,” and “baby sleep,” but future analysis of apps aimed specifically at adolescents is important owing to the ubiquitous use of technology and the high risk for insufficient and delayed sleep in that age range. Our criteria excluding apps with <100,000 downloads may have resulted in missing newer apps that may possibly contain more evidence-based behavioral sleep strategies. Moreover, previous studies that examined sleep apps in adults excluded relaxing music apps, while we chose to include sound or music apps, and, in fact, they made up the majority of the apps examined. Our study did not include apps intended for general use, but we felt it was important to include sound or music apps if they indicated that they were intended to improve children’s sleep and were specifically for bedtime or nighttime purposes. Finally, examination of the developer-provided app description is important since this is information parents may use to help choose which app to use for their children. However, future research is warranted to more comprehensively evaluate children’s sleep apps using an empirically supported rating tools such as the Mobile App Rating Scale [8] and by downloading and user testing the specific features of each app.

### Conclusions

In summary, addressing sleep difficulties in children is important to promote physical, cognitive, and emotional development [1]. Brief behavioral interventions based on learning principles have demonstrated efficacy for children with sleep difficulties [4]. However, families face barriers in accessing evidence-based care owing to a shortage of pediatric sleep specialists and lack of training and knowledge of sleep treatments among non–sleep specialist health professionals [5]. A large variety of sleep apps aimed for use with children exist; yet, the descriptions for each app often do not include evidence-based behavioral sleep strategies. Collaboration between sleep researchers and technology developers may be beneficial for the creation of evidence-supported apps to help with children’s sleep in the future. Additionally, clinicians can support families in selecting apps that align with the evidence base for pediatric sleep.

## References

[1] Byars KC, Yolton K, Rausch J, Lanphear B, Beebe DW (2012). Prevalence, patterns, and persistence of sleep problems in the first 3 years of life. Pediatrics.

[2] Honaker SM, Meltzer LJ (2014). Bedtime problems and night wakings in young children: an update of the evidence. Paediatr Respir Rev.

[3] Meltzer LJ, Johnson C, Crosette J, Ramos M, Mindell JA (2010). Prevalence of diagnosed sleep disorders in pediatric primary care practices. Pediatrics.

[4] Meltzer LJ, Mindell JA (2014). Systematic review and meta-analysis of behavioral interventions for pediatric insomnia. J Pediatr Psychol.

[5] Boerner KE, Coulombe JA, Corkum P (2015). Barriers and facilitators of evidence-based practice in pediatric behavioral sleep care: qualitative analysis of the perspectives of health professionals. Behav Sleep Med.

[6] Grigsby-Toussaint DS, Shin JC, Reeves DM, Beattie A, Auguste E, Jean-Louis G (2017). Sleep apps and behavioral constructs: A content analysis. Prev Med Rep.

[7] Lee-Tobin PA, Ogeil RP, Savic M, Lubman DI (2017). Rate My Sleep: Examining the Information, Function, and Basis in Empirical Evidence Within Sleep Applications for Mobile Devices. J Clin Sleep Med.

[8] Stoyanov SR, Hides L, Kavanagh DJ, Zelenko O, Tjondronegoro D, Mani M (2015). Mobile app rating scale: a new tool for assessing the quality of health mobile apps. JMIR Mhealth Uhealth.

[9] Allen SL, Howlett MD, Coulombe JA, Corkum PV (2016). ABCs of SLEEPING: A review of the evidence behind pediatric sleep practice recommendations. Sleep Med Rev.

[10] Braun V, Clarke V (2006). Using thematic analysis in psychology. Qual Res Psychol.

[11] Leichman ES, Gould RA, Williamson AA, Walters RM, Mindell JA (2020). Effectiveness of an mHealth Intervention for Infant Sleep Disturbances. Behav Ther.

[12] Taki S, Campbell KJ, Russell CG, Elliott R, Laws R, Denney-Wilson E (2015). Infant Feeding Websites and Apps: A Systematic Assessment of Quality and Content. Interact J Med Res.

[13] Zhao J, Freeman B, Li M (2017). How Do Infant Feeding Apps in China Measure Up? A Content Quality Assessment. JMIR Mhealth Uhealth.

[14] Akacem LD, Wright KP, LeBourgeois MK (2018). Sensitivity of the circadian system to evening bright light in preschool-age children. Physiol Rep.

